# Integration of an Image-Based Dietary Assessment Paradigm into Dietetic Training Improves Food Portion Estimates by Future Dietitians

**DOI:** 10.3390/nu13010175

**Published:** 2021-01-08

**Authors:** Dang Khanh Ngan Ho, Wan-Chun Chiu, Yu-Chieh Lee, Hsiu-Yueh Su, Chun-Chao Chang, Chih-Yuan Yao, Kai-Lung Hua, Hung-Kuo Chu, Chien-Yeh Hsu, Jung-Su Chang

**Affiliations:** 1 School of Nutrition and Health Sciences, College of Nutrition, Taipei Medical University, Taipei 110, Taiwan; nganhdk91@gmail.com (D.K.N.H.); wanchun@tmu.edu.tw (W.-C.C.); hysu@h.tmu.edu.tw (H.-Y.S.); 2 Research Center of Geriatric Nutrition, College of Nutrition, Taipei Medical University, Taipei 11031, Taiwan; 3 Department of Obstetrics and Gynecology, Taipei Medical University Hospital, Taipei 110, Taiwan; d119097012@tmu.edu.tw; 4 Department of Dietetics, Taipei Medical University Hospital, Taipei 110, Taiwan; 5 Department of Physical Medicine and Rehabilitation, Taipei Medical University Hospital, Taipei 110, Taiwan; chunchao@tmu.edu.tw; 6 Department of Physical Medicine and Rehabilitation, School of Medicine, College of Medicine, Taipei Medical University, Taipei 110, Taiwan; 7 Department of Computer Science and Information Engineering, National Taiwan University of Science and Technology, Taipei 110, Taiwan; cyuan.yao@csie.ntust.edu.tw (C.-Y.Y.); hua@mail.ntust.edu.tw (K.-L.H.); 8 Department of Computer Science, National Tsing Hua University, Hsinchu 300, Taiwan; hkchu@cs.nthu.edu.tw; 9 Department of Information Management, National Taipei University of Nursing and Health Sciences, Taipei 110, Taiwan; cyhsu@ntunhs.edu.tw; 10 Master Program in Global Health and Development, College of Public Health, Taipei Medical University, Taipei 110, Taiwan; 11 Graduate Institute of Metabolism and Obesity Sciences, College of Nutrition, Taipei Medical University, Taipei 110, Taiwan; 12 Nutrition Research Center, Taipei Medical University Hospital, Taipei 110, Taiwan; 13 Chinese Taipei Society for the Study of Obesity (CTSSO), Taipei 110, Taiwan

**Keywords:** image-based dietary assessment, dietetic training, portion size estimation

## Abstract

The use of image-based dietary assessments (IBDAs) has rapidly increased; however, there is no formalized training program to enhance the digital viewing skills of dieticians. An IBDA was integrated into a nutritional practicum course in the School of Nutrition and Health Sciences, Taipei Medical University Taiwan. An online IBDA platform was created as an off-campus remedial teaching tool to reinforce the conceptualization of food portion sizes. Dietetic students’ receptiveness and response to the IBDA, and their performance in food identification and quantification, were compared between the IBDA and real food visual estimations (RFVEs). No differences were found between the IBDA and RFVE in terms of food identification (67% vs. 71%) or quantification (±10% of estimated calories: 23% vs. 24%). A Spearman correlation analysis showed a moderate to high correlation for calorie estimates between the IBDA and RFVE (*r* ≥ 0.33~0.75, all *p* < 0.0001). Repeated IBDA training significantly improved students’ image-viewing skills [food identification: first semester: 67%; pretest: 77%; second semester: 84%) and quantification [±10%: first semester: 23%; pretest: 28%; second semester: 32%; and ±20%: first semester: 38%; pretest: 48%; second semester: 59%] and reduced absolute estimated errors from 27% (first semester) to 16% (second semester). Training also greatly improved the identification of omitted foods (e.g., condiments, sugar, cooking oil, and batter coatings) and the accuracy of food portion size estimates. The integration of an IBDA into dietetic courses has the potential to help students develop knowledge and skills related to “e-dietetics”.

## 1. Introduction

Magnitudes of estimated errors of traditional self-reported dietary assessment methods such as weighted food records (WFRs) and 24-h dietary recalls (24-HDRs) were observed in various populations including adults [1], adolescents/children [2], obese individuals [3], and athletes [4]. Inaccurate food identification and quantification are significance sources of dietary assessment errors [5]. A diversity of food aids such as real food, food models, two-dimensional (2D) food images, food scales, and a variety of portion size measurement aids (PSMAs) have been developed and incorporated into nutrition educational programs in order to enhance the portion-size estimating skills of the general population and nutrition practitioners [6]. PSMAs include household measuring utensils (e.g., cups, bowls, and spoons), reference objects such as cards or a life-size food atlas, balls (e.g., tennis balls and baseballs), dice, card decks, coins, and parts of the hand (e.g., finger, fist, and palm) [7]. Advantages of PSMAs are their low cost, convenience, availability, and ease of use. Disadvantages include the potential for misreporting dietary intake due to large variations in putative PSMA volumes and a lack of standardization [8]. Recently, Hooper et al. [6] conducted a systematic review evaluating the effects of food aids (e.g., food models, food images, and PSMAs) on food-portion estimation skills. The authors suggested that education with particular food-portion tools may be effective in improving food portion estimation skills among university-recruited participants, and that repeated training was required to maintain those skills over time [6].

The growing availability of smartphones has boosted the development of new technologies that incorporate the use of digital food photography or images for dietary assessments for healthcare applications [9,10]. Smartphone-based food records are reported to be preferable to participants over traditional paper-based dietary assessment methods, as they are easier to incorporate into one’s daily routine [11]. In fact, as early as the 1980s, scientists used food photographs to help subjects assess portion sizes, and food photographs taken of a wide range of individual foods and portion sizes were incorporated into questionnaires for dietary assessments in large epidemiological or population-based longitudinal studies [12]. As a stand-alone dietary assessment method, image-based dietary assessments (IBDAs) are defined as a method that aims to capture all foods and beverages consumed on every eating occasion, and have been used as a primary dietary record [9,13]. The IBDA method has been employed to measure intake levels of energy and nutrients in children [14,15], adults [16,17], and the elderly [18]. A recent meta-analysis and systematic review of the validity of IBDAs showed no statistical difference in energy or macronutrient estimates between IBDA and traditional methods (e.g., WFRs and 24-HDRs); however, IBDAs greatly underestimated energy intake (EI) (−448 kcal) when compared to double-labeled water (DLW), a gold standard method for EI assessments [19]. Those authors concluded that, like traditional methods, image-based methods have serious measurement errors, and more studies are needed to overcome these problems [19].

Food image viewing is a vital skill connecting dieticians to the e-health era. Thus, the ability of dieticians and nutrition practitioners to perform IBDAs is important, as inaccurate food image reviewing may increase measurement errors. Although numerous studies have shown that dieticians and trained human analysts with five years of experience were able to perform IBDAs, few studies have reported on the training dieticians use to develop their digital viewing skills [19]. It is also not known how long it takes to master the necessary skills or how accurate they are. Two recent studies examined the ability of dietetic students and dieticians to review food images, and the authors reported low accuracies in food portion size estimates among American and Australian dietetic students [20] and Malaysian dieticians [21], with respective accuracies of 38% and 24%~32%.

Given the increasing popularity and accessibility of food mobile applications (apps), there is an urgent need to incorporate technology-based teaching strategies into dietetic training programs, as underscored in previous studies [20,21]. To our knowledge, there is no formalized training program that focuses on skills related to IBDAs. Recently, the Ministry of Education, Taiwan, encouraged teaching innovations in higher education such as integrating technology into teaching to improve the digital skills of teachers and students. Hence, we tried to integrate an IBDA into a formal dietetic training program and set up an online IBDA platform as a remedial teaching tool for students who struggle to conceptualize and memorize food portion sizes in the classroom. We hypothesized that integrating IBDAs into formal dietetic training programs would not only improve students’ portion size estimation accuracy, but also their digital food viewing skills. The broad aims were to: (1) train dietetic students to take standardized food images and perform IBDAs, (2) assess students’ abilities to perform food identification and quantification using food images, and (3) evaluate students’ receptiveness and response to IBDA integration.

## 2. Materials and Methods

### 2.1. Participants

Participants were second-year undergraduate students, aged ≥19 years, and enrolled in a nutritional practicum (NP) course in the School of Nutrition and Health Sciences, Taipei Medical University (TMU) (Taipei, Taiwan), between September 2018 and July 2019. Eighty-four students participated in the first-semester study (September 2018 to January 2019), and 81 students joined the second-semester study (February to July 2019). In total, 81 students completed both the first- and second-semester studies; three students only completed the first semester study. All students completed the online pretest. The study protocol was approved by the Institutional Review Board of TMU (N201904035), which covered the study period between first and second semesters.

### 2.2. Study Design

#### 2.2.1. Integration of an IBDA to the Formal Dietetic Training Program

**Figure 1:**
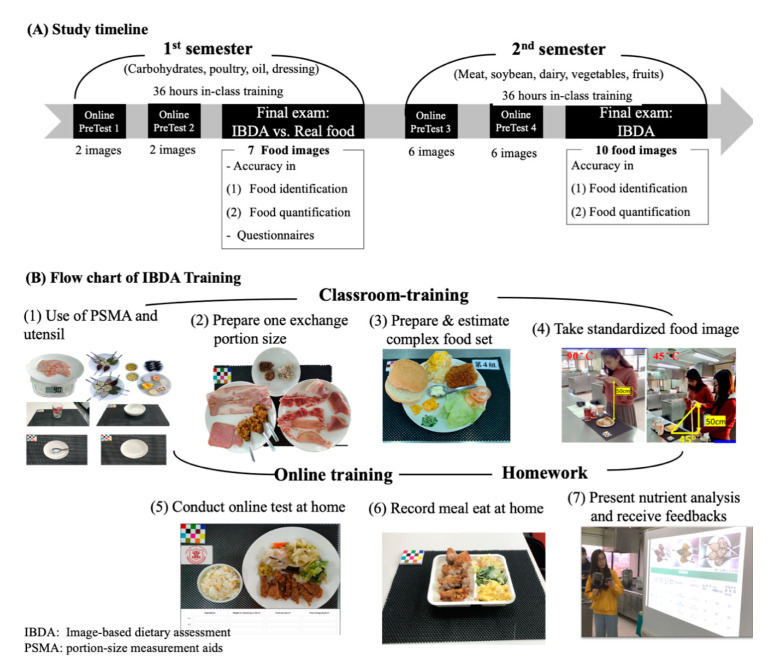
Flowchart and timeline of the integration of the image-based dietary assessment (IBDA) training protocol into the nutrition practicum course. (**A**) Study timeline; (**B**): Flowchart of IBDA training. PSMA: portion-size measurement aids.

The NP course is a kitchen-based laboratory course which teaches practical skills involved in dietary assessments such as culinary skills, food classification, and food portion size estimation. Traditionally, students were trained to estimate food portion sizes based on real food visual estimations (RFVEs) and estimated portion sizes and calories using PSMAs, utensils, and food scales. We integrated IBDA training into the NP course in parallel with real food training in the October 2018 to July 2019 academic year. The NP course is a one-year course which consists of 18 lessons covering a wide variety of foods: carbohydrates and sugar, meat (poultry, red meat, fish, and seafood), oils and dressings, dairy, eggs, vegetables, and fruits. Figure 1A shows the overall study timeline of IBDA training. Briefly, we aimed to explore the level of agreement among students’ estimates when applying the two methods (RFVE and IBDA) and their receptiveness and response to the IBDA training in the first semester. In the second semester, we investigated whether students’ IBDA performance had improved and been maintained over the one-year training period.

Figure 1B shows a flowchart of the IBDA training protocol. Students were trained to prepare food according to the recipient and take standardized food images depicting a range of commonly consumed portion sizes. Factors such as image angle, height and width, thickness and depth of food on a plate or depth in a bowl may influence the perception of students when performing an IBDA [12]. In the first class of the first semester, research staff demonstrated how to use PSMAs, utensils, and food scales (HD-SK 8001, Hon Der precision scale, Taipei, Taiwan) to prepare one exchange portion size (Taiwan portion size unit) and take standardized food images. For a complex food set, food ingredients were separated and individually weighed using the food scale; hence, the quantities and identities of the foods in the complex food image were known (ground truth). Complex food sets were purchased from restaurants/vendors or prepared in the kitchen using standardized recipes. Foods (single food or complex food set) were placed on a standardized plate (small: 17 × 17 cm; large: 26 × 26 cm) or bowl (small: 13 × 7.5 cm; medium: 16.5 × 7.5 cm; large: 22 × 8 cm) and drinks in a standardized cup (width 10 cm × height 16 cm). Plates or bowls were then placed on a table mat (30 × 45 cm) with a fiducial marker (4.5 × 5.5 cm) as a reference object for interpreting the color and size of the foods in the image [20,22]. Food images were taken using the AngleCam application (© Derekr Corp.—Google Play, Taipei, Taiwan), which is a scientific camera that gives an accurate angle of a picture. One image was taken at a camera angle of approximately 90° and a distance of 50 cm above the table mat, and another was taken with a camera angle of approximately 45° and a 50-cm height. The food photographs were uploaded into the e-learning system for that class. 

There were two off-school assignments for students. Firstly, students estimated food portion sizes in the food images taken in the class and presented the results in the next class session. The principal investigator corrected and gave feedback on each presentation. Secondly, students were required to complete a total of four online IBDA pretests (two per semester) (Figure 1A). An online pretests platform was created as a remedial teaching tool for students who were struggling to conceptualize and memorize food portion sizes from images. The pretests were created using the LimeSurvey (LimeSurvey, vers. 3.23.7, Hamburg, Germany) as extra exercises for students to practice portion size estimations from photographs at home, and data were analyzed as the pretest results. For each semester, we provided two online pretests prior to the final exam for students to practice IBDA. In the pretests, students were asked to report: (1) names of the presented food items in each image, (2) food portion size (weight and exchange), and (3) total number of calories in each food image.

#### 2.2.2. Agreement between Food Images and Real Food Visual Estimations (RFVEs)

In the first semester, we evaluated students’ abilities to identify foods and estimate portion sizes between the IBDA and RFVE for the first time. Seven food sets (buttered toast, sugar-sweetened coix-seed beverage, red bean dorayaki, black pepper sauce noodles, chicken sandwich, sweet corn, and sweet potato) were prepared, and the ground truth weight of each food set was recorded with a food scale by a registered dietitian. The dietitian calculated the portion sizes (as exchange of Taiwan standard servings) and total calories according to the ground truth weight of each food set. Standardized food images (at 45° and 90°) of those food sets were taken for the IBDA test prior to the exam. In both the IBDA and RFVE exams, students were asked to report all food ingredients and quantify their ground truth weights, exchange, and total calories of each food set. After the exam, students completed a brief questionnaire to assess their receptiveness and response to the IBDA training and difficulties encountered in reviewing food images. The questionnaire was modified based on a study by Howes et al. [20]. For example, students were asked to comment on a series of multiple-choice questions concerning problems/difficulties related to identifying and quantifying foods from images. Students reported their level of agreement of integration of IBDA training into the NP course using a 10-point scale (with 1 as strongly agree and 10 as strongly disagree).

#### 2.2.3. Accuracy of the IBDA

The food images were selected for the IBDA using the following criteria: they are the most commonly consumed foods in Taiwan and they represent a variety of food categories which are taught in the class. In the test, students were asked to report: (1) names of the food items presented in each food image, (2) food portion sizes (weight and exchange), and (3) total calories of each food set. We also evaluated whether repeated IBDA training improved their identification and quantification of omitted food items. For this purpose, in the pretest, students were trained to identify foods that were omitted in the first semester (e.g., condiments, sugar, cooking oil, or batter coatings), and each student’s performance was re-evaluated in the second semester. The effects of repeated IBDA training on those who had performed most poorly in the first semester [defined as ≥3 answers within >50% of the ground truth kcal (*n* = 14)] were also evaluated.

#### 2.2.4. Definition of Food Identification and Quantification

a. Accuracy of food identification

Food ingredient identification was categorized into “accurate”, “inaccurate”, and “omitted”. For students who failed to recognize a food ingredient from an image, the provided answer was coded as “omitted”. “Accurate” was defined if the answer matched the standard food name. For participants who reported wrong food items in an image, the provided answer was regarded as “inaccurate”.

We defined the accuracy of food identification as follows:

(1) % accurate food items = 100 × (total “accurate” number of food items identified by participants/total actual number of food items);
(2) % inaccurate food items = 100 × (total “inaccurate” number of food items reported by participants/total actual number of food items); and
(3) % omitted food items = 100 × (total number of “omitted” food items by participants/total actual number of food items).

b. Accuracy of food quantification

To calculate the accuracy of food quantification, the food weight/kcal estimated by students was compared to the ground truth weight/kcal. The percentage difference between the estimated and actual weight/kcal was calculated using this formula:

(1) $$\frac{\left(\mathrm{estimated}\;\mathrm{weight}\;\mathrm{or}\;\mathrm{kcal}-\mathrm{ground}\;\mathrm{truth}\;\mathrm{weight}\;\mathrm{or}\;\mathrm{kcal}\right)}{\mathrm{ground}\;\mathrm{truth}\;\mathrm{weight}\;\mathrm{or}\;\mathrm{kcal}}\times \;100\%$$

An “accurate” estimation was defined as an estimate which fell within ±10% difference of the ground truth weight or kcal. Estimates with >+10% were regarded as “overestimates”, whereas estimates with <−10% were regarded as “underestimates”. “Omitted” was defined if students failed to quantify food items in an image. Calories of each food item (ground truth kcal) were estimated from the actual weight (g) based on the Taiwanese Food Composition and Nutrient Database.

c. Percentage estimated error

The percentage estimated error of each student’s estimate for each food was computed in both absolute values and a difference method [5] using the following formula:

(2) $$\frac{\left(\mathrm{estimated}\;\mathrm{weight}\;\mathrm{or}\;\mathrm{kcal}-\mathrm{ground}\;\mathrm{truth}\;\mathrm{weight}\;\mathrm{or}\;\mathrm{kcal}\right)}{\mathrm{ground}\;\mathrm{truth}\;\mathrm{weight}\;\mathrm{or}\;\mathrm{kcal}}\times \;100\%$$

Absolute values of the estimated error were calculated and classified into six groups (within 10%, 20%, 30%, 40%, 50%, and >50%) in order to evaluate the accuracy of the estimates. The effectiveness of the training was evaluated by examining changes in the absolute percentage error for all students and for all food items from the first to the second semester. The difference method (actual values) was used to distinguish between overestimates (positive scores) and underestimates (negative scores) for each food image.

### 2.3. Data Analysis

An analysis was conducted using GraphPad Prism 5 (GraphPad Software, La Jolla, CA, USA) and SPSS software (version 23.0, IBM Corp., Armonk, NY, USA). The Kolmogorov-Smirnov test was used to determine whether the data were normally distributed. Normally distributed data are presented as the mean and 95% confidence interval (CI) and median and interquartile range (IQR) [quartile 1 (Q1); Q3] for nonparametric data. Spearman’s coefficient was used to obtain correlations between students’ calorie estimates for each food item with two methods (RFVE vs. IBDA) with coefficients of >0.5 and >0.7 respectively indicated moderate and high degrees of correlation. Cohen kappa and cross-classification tests were performed to evaluate the interrater agreement between the accuracy the estimates from RFVE and IBDA. This was analyzed by calculating the chance of misclassification between the two methods (e.g., a student estimate being classified in the 10% accuracy group by RFVE but classified in the 20% accuracy by IBDA or vice versa). Kappa k values ≤ 0 as no agreement, 0.01–0.20 as none to slight agreement, 0.21–0.40 as fair, 0.41–0.60 as moderate, 0.61–0.80 as substantial, and 0.81–1.00 as almost perfect agreement [23]. The Kruskal-Wallis One-way ANOVA and linear trend test was used to evaluate the effects of IBDA pretest training and overall accuracy of the final test using the median value of each semester data. The significance level was set to *p* < 0.05.

## 3. Results

Participants (92%) were largely female (83%), second-year undergraduate students (aged 20~22 years) in Nutrition and Health Sciences (92%), or with a double major (8%) in Nutrition and Public Health, Food Safety, Nursing, or Gerontology Health Management. Eighty-four students participated in the first semester test and the subsequent questionnaire survey. Three students did not enroll in the second semester course; as a result, only 81 students completed both the first and second semester exams. All participants completed the online IBDA pretests.

### 3.1. Accuracy of Food Identification and Quantification between the IBDA and RFVE

In the first semester, we compared students’ ability to perform food identification and quantification based on food images (IBDA) and real food (RFVE) (Table 1). On the food tested, 64% of participants were able to correctly identify the ingredients based on images and 71% for the RFVE. No differences were observed in portion size estimations (within ±10% difference of total calories) between the IBDA (23%) and RFVE (24%) (Table 1). However, students tended to underestimate food portion sizes when using the IBDA (50%) but overestimated them in the RFVE (36%). Omissions were highest for batter coatings (IBDA: 95%, RFVE: 99%), mayonnaise (IBDA: 92%, RFVE: 93%), sauces (IBDA: 82%, RFVE: 43%), oils (IBDA: 52%, RFVE: 71%), and sugars (IBDA: 48%, RFVE: 41%). Students tended to underestimate sweet corn (IBDA: 67%, RFVE: 52%) and sweet potato (IBDA: 86%, RFVE: 85%), but overestimated chicken filling in a sandwich (IBDA: 93%, RFVE: 73%), butter (IBDA: 52%, RFVE: 65%), vegetables (IBDA: 59%, RFVE: 79%), and coix-seed beverage (IBDA: 50%, RFVE: 70%). The Spearman correlation analysis showed weak (dorayaki: *r* = 0.39; oil: *r* = 0.40; coix-seed beverage: *r* = 0.33), moderate (noodles: *r* = 0.68; sweet corn: *r* = 0.64; sweet potato: *r* = 0.59; egg: *r* = 0.62; butter: *r* = 0.63; vegetables *r* = 0.63; red bean: *r* = 0.57 and chicken filling: *r* = 0.52), and strong correlations (toast: *r* = 0.76 and sugar: *r* = 0.71) between the IBDA and RFVE. Kappa statistics (Table 1) showed that sugar had the strongest agreement (κ = 0.95, *p* < 0.0001), following moderate agreements on sweet corn (κ = 0.531, *p* < 0.0001), sweet potato (κ = 0.502, *p* < 0.0001), toast (κ = 0.575, *p* < 0.0001), egg (κ = 0.498, *p* < 0.0001), butter (κ = 0.498, *p* < 0.0001) and coix seed beverage (κ = 0.457, *p* < 0.0001), and fair agreements on red bean (κ = 0.248, *p* = 0.001) and oil (κ = 0.318, *p* = 0.03).

### 3.2. Student Receptiveness and Response to IBDA Integration

Table 2 summarizes student receptiveness and response to IBDA training. As to the food tested for food identification, 70% of students found the IBDA more difficult than the RFVE (2%). A similar rate for food quantification was seen (68% for the IBDA and 30% for the RFVE). Factors that affected IBDA performance included the following: foods being mixed (65%) or food ingredients being hidden inside (36%), lacking the ability to estimate food portion sizes (52%), the angle at which the food image was taken (44%), food presentation (26%), and food images looking different from real food (21%). On a scale of 0 to 10, moderate to strong agreement (7.0~8.5 points) was found as to the usefulness of integrating the IBDA into training or as an important dietary assessment method (Table 2).

### 3.3. Overall Performance of the IBDA

**Figure 2:**
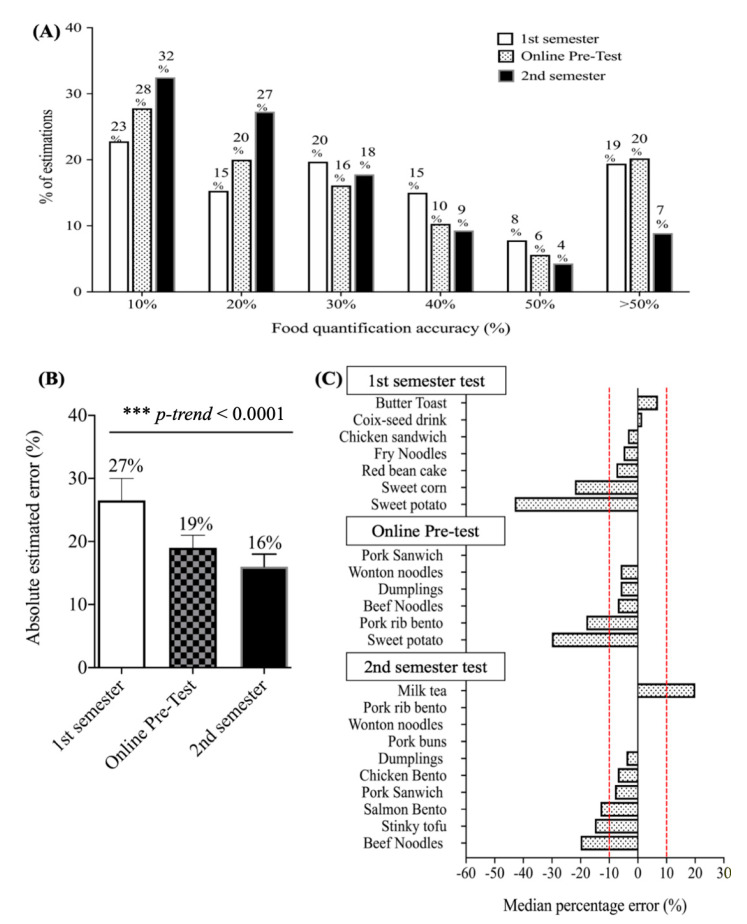
Students’ overall performance of total calorie estimations (**A**); The absolute estimated error (**B**); data are presented as the median with the 95% confidence interval (CI); linear trend test using the median value of each semester data; *** *p*-trend < 0.0001 (**C**) median estimated error of total calories in the first semester (*n* = 84), the online pretest (*n* = 74), and the second semester (*n* = 81) of the nutrition practicum course.

Table 3 shows a steady improvement in the IBDA performance in food identification and quantification across the first semester, pretest, and second semester. As for the food tested for food identification, the accuracy increased over time, from 67% in the first semester and 77% in the pretest to 84% in the second semester (Table 3). When the error margin of estimated calories was within ±20%, the accuracy of food quantification rose from 38% in the first semester and 48% in the pretests, to 59% in the second semester (Figure 2A). Estimated calories within ±10% also increased (first semester: 23%, pretest: 28%, and second semester: 32%) (Figure 2A, Table 3). In contrast, the proportion of >±50% of the estimated error decreased from 19% and 20% to 7%, respectively (Figure 2A). Improvement in accuracy of food quantification also resulted in an overall reduction in the absolute estimated error of calories of 27%, 19%, and 16% for the first semester, pretest, and second semester, respectively (Figure 2B). Figure 2C shows that estimated calories of “sweet corn” [−22% (−22%; 4%)], “sweet potato (first semester)” [−43% (−57%; −14%)], “pork rib bento (pretest)” [−18% (−34%; −6%)], “beef noodles (second semester)” [−20% (−31%; −5%)], “stinky tofu” [−15% (−35%; −4%)], and “salmon bento” [−13% (−20%; −5%)] were greatly underestimated with the highest median percentage error and IQR. In contrast, sugar-sweetened milk tea was considerably overestimated [+20% (−5%; 80%)]. Table 3 shows the tendency of underestimation of estimated calories (overall: 46%) and overestimation (overall: 23%), by the IBDA (Table 3).

### 3.4. Effects of Repeated IBDA Training

**Figure 3:**
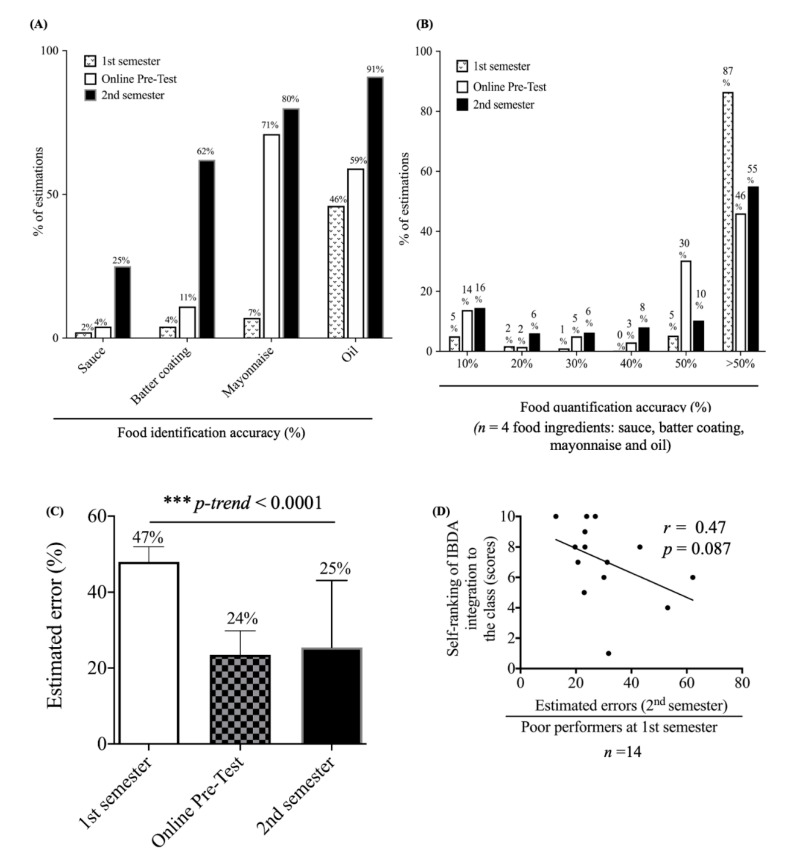
Effects of repeated image-based dietary assessment (IBDA) training on the identification (**A**) and quantification (**B**) of condiments, cooking oils, and batter coatings, and poor performers (defined as ≥3 answers within >50% of the ground truth kcal on the first semester exam) (*n* = 14); linear trend test using the median value of each semester data; *** *p*-trend < 0.0001 (**C**). Spearman correlation analysis of self-ranking scores of usefulness of IBDA integration into the course and the median estimated error among poor performers (*n* = 14) (**D**).

Because students tended to omit condiments (e.g., sauces, mayonnaise, and dressings), cooking oil, and batter coatings of deep-fried pork and chicken (Table 1, first semester), we next evaluated the effects of repeated training on those foods. Figure 3A shows the overall improvements in food identification of sauces (first semester: 2%, pretest: 4%, second semester: 25%), batter coatings of deep-fried foods (first semester: 4%, pretest: 11%, second semester: 62%), mayonnaise (first semester: 7%, pretest: 71%, second semester: 80%), and cooking oils (first semester: 46%, pretest: 59%, second semester: 91%). However, in the test of food quantification, the training only increased a small proportion of the best performers (within ±10% of estimated calories: first semester: 5%, pretest: 14%, second semester: 16%) and reduced the number of the poorest performers (>±50% of estimated calories: first semester: 87%, pretest: 46%, second semester: 55%) (Figure 3B). Figure 3C shows that for the poorest performers on the first semester exam (*n* = 14), repeated training decreased the estimated errors from 47% in the first semester to 25% on the second semester test. A weak inverse relationship between the median estimated error and self-ranking score of usefulness of IBDA integration into the course was found (Figure 3D).

## 4. Discussion

Despite the fact that most technology-based dietary assessment studies employed dieticians/nutritionists to perform IBDAs [19], little research has explored how dieticians develop their “tele-dietetic” knowledge, or how accurate their IBDA skills are. Our study showed that after repeated training, more than half (59%) of junior students were able to estimate calories within ±20%, and most importantly, that the accuracy of those who were the poorest performers improved. Specifically, repeated IBDA training gradually improved dietetic students’ digital food-viewing skills [food identification (first semester: 67%, pretest: 77%, second semester: 84%) and quantification (±10%: first semester: 23%, pretest: 28%, second semester: 32%)] and reduced the proportion of the poorest performers from 27% to 11% (>±50% of estimated calories). The overall performance rate was similar to the image-viewing skills of Australian and American dietetic students, at 79.5% and 38% accuracy for food identification and quantification (defined as within ±10%), respectively. A similar food image portion size accuracy (±10%: 24%~32%) was also reported for nutrition professionals in Malaysia [21]. We observed a weak inverse correlation between the receptiveness to IBDAs and the estimated errors of food portion sizes among those who were the poorest performers (*r* = 0.47, *p* = 0.087; *n* = 14). This suggests that increased receptivity to IBDA training may improve a student’s performance, even among low performing students. This finding is encouraging, since to our knowledge, there is no formalized training program related to skills of IBDAs, and only a limited number of studies have investigated the digital food-viewing skills of dietetic students.

Interestingly, a higher proportion of students (68%~70%) thought that the IBDA was more difficult than RFVE, but their performance in food identification and quantification tests did not differ. Nelson et al. [9] proposed that the accuracy of food photograph-based dietary assessments includes perception, conceptualization, and memory. This principle applies to both RFVEs and IBDAs, except that RFVEs are 3D and images are 2D. Traditionally, RFVEs are key nutritional education in NP courses, which are kitchen-based to provide hands-on training in food preparation, cooking, and culinary skills, and help students perceive diverse food types and portion sizes. Real food is 3D and is thought to be more advantageous over 2D food images in conceptualizing food portion sizes and hence, helping students memorize the diverse range of food portion sizes, especially amorphous foods (with irregular food shapes). The current results showed similar accuracies between RFVEs and IBDAs, and results of calorie estimations from these two methods were moderately to strongly correlated (Table 1). This finding is in agreement with an earlier study by Williamson et al. [24]. Food images that students perceived to be the most difficult to estimate were those with the highest estimation errors and omission rates (sauces, sugar, and drinks). This indicates that a student’s perception generally corresponds to actual estimation errors. Another interesting finding is that students tended to underestimate food portion sizes with the IBDA while overestimating them with the RFVE. As students expressed difficulty in conceptualizing the volume of 2D food images, future study is needed to investigate whether the incorporation of 3D food images can improve the accuracy of IBDAs.

Effective education about food-portion sizes is a fundamental skill of nutrition practitioners [6]. Training, especially repeated training, is known to reduce measurement errors and improve the accuracy of portion size estimates. The literature shows that training improves the accuracy of food portion size estimates among non-nutrition-major university students [6], nutrition-trained university students [20], dietetic students [5,20], and nutrition professionals [21]. Training also affects the accuracy of IBDAs. The need to improve digital food-viewing skills among dietetic students and registered dieticians was underscored in previous studies [20,21]. Howes et al. acknowledged the lack of formalized training for skills related to IBDA in the university, and as a first attempt, those authors investigated undergraduate dietetic students’ abilities and challenges encountered when reviewing food images [20]. They observed that both “training-related factors” (e.g., culinary skills, experience with food preparation, food labs, volume measurements, and searching food databases) and “technology-related factors” (e.g., poor food images, angle of food images, size of the screen, use of fiducial markers, and hidden or mixed food ingredients) affected dietetic students’ digital food-viewing skills [20]. To minimize the bias of “technology-related factors” on the accuracy of portion size estimates, we taught students how to take standardized food images (e.g., from two angles using fiducial markers and standardized utensils). However, technology-related factors cannot resolve inherent human measurement errors. Our students agreed that cooking experience, watching gourmet shows, and being familiar with recipes can improve their dietary assessment skills. This suggests that culinary training is still a key element in reducing human measurement errors regardless of the type of measurement tools (e.g., real food or IBDAs) [25,26].

Another concern is the variation in methods used to estimate the accuracy of food portion sizes [6]. One-third (32%) of our junior undergraduate dietetic students could accurately estimate food portion sizes within 10% of the actual kcal. This result was higher than the 18.5% of accurate estimates reported by Japer et al. among nutrition-major students [27], and similar to nutrition professionals in Malaysia (24%~33% within 10% of total estimates) [21], but slightly lower than US and Australian dietetic students (38% within 10% of total estimates) [20]. However, it should be noted that participants in the current study were junior dietetic students, and students had received no training on food estimation skills before the NP course. In contrast to studies such as ours and those of Japer [27], Howes [20], and Fatehah et al. [21] which reported results as accuracies (±10%), the majority of studies report results as percentages of estimated errors which were calculated based on the absolute (pooled results as a positive error, i.e., which does not distinguish between over- or under- estimations) or difference methods (defined as accuracy within ±10%, and then reporting overestimations as positive errors and underestimations as negative errors) [6]. For example, Aroyo et al. [5] reported that real food training improved median absolute estimation errors from 64% (untrained) to 53% (trained) among nutrition-trained university students. Bolland and colleagues showed that food model training improved mean absolute estimation errors from 92.4% (untrained) to 65.7% (trained) [28] and 94.0% (untrained) to 58.7% (trained) in the general population [29]. Individual overestimation (positive) and underestimation (negative) results may cancel each other out, hence lowering the overall effect [6]. This also makes overall training results more difficult to comprehend or interpret. For example, our study showed that estimated errors decreased to 16% but the overall accuracy rate was still low (32% within 10%).

The complexity of food types used in the IBDA test also affected students’ performance. In line with previous findings [20,21,27], students generally experienced difficulties with amorphous foods (e.g., sweet potato and red bean paste), beverages (e.g., coix-seed drink and milk tea), batter coatings of deep-fried chicken or pork, vegetables (lettuce, salads, and cabbage), and hidden or unseen ingredients (sauces, sugar, cooking oil, butter, and mayonnaise). These errors were not restricted to students or by the methodology. Trained dieticians also experience difficulty in quantifying those foods, whether using real foods [5,27] or food images [20,21,30,31]. Omissions are likely to explain the inaccuracy of portion size estimations. We found that both the IBDA and RFVE had high omission rates in condiments, and hidden or unseen energy-dense foods. Dietary fats like cooking oils and sugar in drinks are invisible in both real food and digital images, and calories from oils, dressings, and sauces tend to be neglected by inexperienced dieticians [32]. The current study showed that repeated training greatly improved the identification of condiments, cooking oils, and mayonnaise, but increased food identification did not result in a higher accuracy of calorie estimations (Figure 3A,B). This finding is consistent with previous studies, which suggested that food items with an irregular shape or lower energy density such as vegetables tended to be underestimated [33,34]. We also noted that students tended to underestimate amorphous foods (e.g., sweet potato) and vegetables (e.g., salads and sandwich fillings such as lettuce) in both the RFVE and IBDA. The overall tendency of underestimation was greater in the IBDA than the RFVE (50% vs. 25%, Table 1).

### Strengths and Limitations

The strengths of this study include the high completion rate (100%), longitudinal repeated training (nine months), a moderate number of dietetic students who participated in the novel program, and the direct comparisons we made between traditional and technology-based dietary assessment methods (RFVE and IBDA). To reinforce students’ conceptualization of food portion sizes, the present work established a series of off-campus, online practices as a remedial teaching platform. The current study also included a wide range of images of the most commonly consumed Taiwanese foods, which represents real habitual food intake and standard portion sizes. Limitations include the generalizability of the students (from one university) and findings that cannot be applied more broadly, as we only assessed junior dietetic students (second-year undergraduate students), restrictions of the exam time, and a lack of a control group for the IBDA. The limited sample size highlights the need for future collaboration in multiple dietetic schools in order to recruit a large-scale representative population. The validity of the current study findings also needs to be confirmed in free-living settings.

## 5. Conclusions

Repeated IBDA training was shown to improve the digital dietary assessment skills of dietetic students; however, innovative technologies to assist human analysts to reduce measurement errors of the IBDA are also needed. Further research is also encouraged to unravel questions of how to implement “e-dietetics” in dietetics training programs and how to improve students’ digital food-viewing skills for the future eHealth era.

## Figures and Tables

**Table 1:** Accuracy in food identification and portion size quantification in image-based dietary assessments (IBDAs) and real food visual estimations (RFVEs) evaluated in the first semester of the nutrition practicum course (*n* = 84).

Food Item	IBDA					RFVE					Spearman Correlation	Cohen Kappa ^f^
	Identified Correctly (%) ^a^	Quantified Correctly ± 10% (%) ^b^	Overestimated (%) ^c^	Underestimated (%) ^d^	Omitted (%) ^e^	Identified Correctly (%)	Quantified Correctly ± 10% (%)	Overestimated (%)	Underestimated (%)	Omitted (%)	*r*; *p*-Value	*k*; *p*-Value
Sweet corn	100%	15%	19%	67%	0%	100%	21%	26%	52%	0%	0.638; *p* < 0.0001	0.531; *p* < 0.0001
Sweet potato	87%	8%	6%	86%	0%	93%	7%	8%	85%	0%	0.592; *p* < 0.0001	0.502; *p* < 0.0001
Noodles	91%	26%	30%	44%	0%	91%	21%	45%	34%	0%	0.679; *p* < 0.0001	0.0523; *p* = 0.292
Dorayaki	98%	12%	60%	26%	0%	88%	18%	43%	39%	0%	0.393; *p* < 0.001	0.267; *p* < 0.0001
Toast	97%	52%	28%	18%	2%	93%	38%	47%	14%	7%	0.755; *p* < 0.0001	0.575; *p* < 0.0001
Eggs	94%	76%	15%	8%	0%	100%	93%	5%	2%	0%	0.624; *p* < 0.0001	0.498; *p* < 0.0001
Chicken	95%	1%	93%	2%	0%	90%	2%	73%	15%	10%	0.519; *p* < 0.0001	0.041; *p* = 0.354
Butter	71%	10%	52%	21%	17%	88%	7%	65%	27%	0%	0.628; *p* < 0.0001	0.498; *p* < 0.0001
Red beans	91%	17%	48%	32%	4%	96%	18%	56%	23%	4%	0.572; *p* < 0.0001	0.248; *p* = 0.001
Mayonnaise	7%	4%	2.40%	2.40%	92%	10%	5%	1%	1%	93%	NA	NA
Batter coating	4%	1%	1.20%	2.40%	95%	1%	0%	0%	1%	99%	NA	NA
Vegetables	76%	8%	59%	13%	19%	100%	5%	79%	16%	0%	0.632; *p* < 0.0001	0.035; *p* = 0.567
Sugar	51%	6%	18%	29%	48%	54%	5%	22%	32%	41%	0.706; *p* < 0.0001	0.95; *p* < 0.0001
Sauce	2%	0%	12%	6%	82%	44%	1%	38%	18%	43%	NA	NA
Oil	46%	15%	23%	10%	52%	26%	8%	4%	17%	71%	0.404; *p* = 0.004	0.318; *p* = 0.03
Coix seed beverage	69%	2%	50%	15%	32%	69%	5%	70%	20%	5%	0.331; *p* = 0.0073	0.457; *p* < 0.0001
Overall	67%	23%	28%	50%		71%	24%	36%	25%			

^a^ Percentage of students correctly identifying food items. ^b^ Percentage of students quantifying food calories within ± 10% of ground truth calories. ^c^ Overestimate: >10% of the ground truth total kcal; ^d^ Underestimate: <−10% of the ground truth total kcal; ^e^ Omitted: students who failed to recognize and quantify food items from images. ^f^ Kappa for students’ estimate in categories of accuracy (within 10%, 20%, 30%, 40%, 50% and >50%).

**Table 2:** Student receptiveness and response to the image-based dietary assessment (IBDA) (*n* = 84).

	Percentage (%)
Which method was more difficult to identify food items	
- Real food visual estimation (RFVE)	2% (2/84)
- IBDA	70% (59/84)
- Both	27% (23/84)
Which method was more difficult to quantify food items	
- RFVE	2% (2/84)
- IBDA	68% (57/84)
- Both	30% (25/84)
What challenges did you experience when trying to identify food items in the images?	
- The way the food was placed made it difficult to evaluate.	26% (22/84)
- Food pictures were too different from real foods.	21% (21/84)
- The food was mixed together making it difficult to recognize.	65% (55/84)
What was the most challenging aspects of estimating the quantity of the food items in the images?	
- The angle at which the picture was taken made it difficult to judge the size of the food.	44% (37/84)
- It was impossible to estimate the portion size of hidden food items.	36% (30/84)
- A student’s ability to estimate the portion size was not related to the food image itself.	52% (44/84)
Students’ responses to the integration of the IBDA into the course using a 10-point Likert scale	Mean score ± SD
- IBDA training improved your food identification skills.	7.1 ± 2.3
- IBDA training improved your food quantification skills.	8.5 ± 2.1
- IBDA training should be integrated into the dietetic training program.	7.0 ± 2.6
- IBDA is an important method of dietary assessment.	8.1 ± 3.2

**Table 3:** Students’ overall performance of food identification and calorie quantification in the first semester (*n* = 84), in the online pretest (*n* = 74), and in the second semester (*n* = 81) of the nutrition practicum course.

Food Image	Number of Participants	Ground Truth Total Kcal	Food Identification Accuracy (%) ^a^	Estimated Total Kcal	Estimated Error (%) ^a^	Accurate (%) ^b^	Overestimated (%) ^c^	Underestimated (%) ^d^
				Median [Q1; Q3]	Median [Q1; Q3]			
First semester test								
Sweet corn	84	134	100%	105 [105; 140]	−22 [−22; 4]	14%	19%	67%
Sweet potato	84	324	87%	186 [140; 280]	−43 [−57; −14]	8%	6%	86%
Buttered toast	84	294	85%	315 [262; 344]	7 [−11; 17]	44%	31%	25%
Red bean cake	84	358	95%	332 [244; 420]	−8 [−32; 17]	19%	32%	49%
Chicken sandwich	84	402	80%	388 [292; 485]	−4 [−28; 1]	24%	33%	43%
Fried noodles	84	465	55%	444 [315; 545]	−5 [−32; 17]	26%	30%	44%
Coix-seed beverage	84	198	69%	201 [153; 260]	2 [−23; 31]	24%	42%	35%
Overall			67%			23%	28%	50%
Online Pretest								
Sweet potato	74	308	92%	215 [140; 280]	−30 [−55; −9]	23%	4%	64%
Dumplings	74	560	90%	541 [416; 665]	−28 [−6; 15]	25%	26%	39%
Wonton noodles	74	640	76%	607 [511; 701]	−6 [−25; 7]	31%	23%	38%
Beef noodles	74	808	89%	685 [509; 854]	−7 [−28; 6]	34%	15%	42%
Pork sandwich	74	552	78%	532 [360; 608]	0 [−17; 4]	34%	24%	30%
Pork rib bento	74	853	75%	696 [557; 807]	−18 [−34; −6]	20%	9%	59%
Overall			77%			28%	17%	45%
Second semester test								
Dumplings	81	560	95%	555 [472; 679]	−4 [−19; 18]	30%	31%	40%
Buns	81	389	99%	388 [313; 487]	0 [−10; 25]	28%	37%	35%
Pork sandwich	81	552	95%	510 [429; 573]	−8 [−22; 4]	41%	14%	46%
Stinky tofu	81	392	75%	334 [255; 378]	−15 [−35; −4]	21%	20%	59%
Wonton noodles	81	640	76%	640 [561; 729]	0 [−13; 14]	30%	36%	35%
Beef noodles	81	808	99%	650 [564; 769]	−20 [−31; −5]	23%	11%	65%
Pork bento	81	853	80%	850 [771; 920]	0 [−10; 8]	57%	21%	22%
Salmon bento	81	992	82%	864 [797; 942]	−13 [−20; −5]	35%	7%	58%
Chicken bento	81	934	78%	857 [770; 949]	−7 [−17; 3]	42%	11%	47%
Milk tea	81	119	89%	143 [114; 214]	20 [−5; 80]	19%	60%	21%
Overall			84%			32%	25%	43%

Data are presented as the median [first quantile (Q1); third quantile (Q3)]. ^a^ The estimated error was calculated as: percentage of difference (%) = (visual estimated kcal − ground truth kcal) × 100%/ground truth kcal. ^b^ Accurate: a student’s estimate was within ±10%, ±20%, or ±30% of the ground truth total kcal; ^c^ Overestimated: >10% of the ground truth total kcal; ^d^ Underestimated: <−10% of the ground truth total kcal.

## Data Availability

The datasets used and/or analyzed during the current study are available from the corresponding author on reasonable request.
